# Interleukin-18 and fibroblast growth factor 2 in combination is a useful diagnostic biomarker to distinguish adult-onset Still’s disease from sepsis

**DOI:** 10.1186/s13075-020-02200-4

**Published:** 2020-05-07

**Authors:** Tomohiro Koga, Remi Sumiyoshi, Kaori Furukawa, Shuntaro Sato, Kiyoshi Migita, Toshimasa Shimizu, Masataka Umeda, Yushiro Endo, Shoichi Fukui, Shin-ya Kawashiri, Naoki Iwamoto, Kunihiro Ichinose, Mami Tamai, Hideki Nakamura, Tomoki Origuchi, Fumiaki Nonaka, Akihiro Yachie, Hideaki Kondo, Takahiro Maeda, Atsushi Kawakami

**Affiliations:** 1 Department of Immunology and Rheumatology, Division of Advanced Preventive Medical Sciences, Nagasaki University Graduate School of Biomedical Sciences, Nagasaki, Japan; 2 Center for Bioinformatics and Molecular Medicine, Nagasaki University Graduate School of Biomedical Sciences, 1-12-4 Sakamoto, Nagasaki, 852-8523 Japan; 3 Clinical Research Center, Nagasaki University Hospital, Nagasaki, Japan; 4 Department of Rheumatology, Fukushima Medical University School of Medicine, Fukushima, Japan; 5 Department of Community Medicine, Nagasaki University Graduate School of Biomedical Sciences, Nagasaki, Japan; 6 Department of Internal Medicine, Sasebo City General Hospital, Sasebo, Japan; 7 Department of Pediatrics, School of Medicine, Institute of Medical, Pharmaceutical and Health Sciences, Kanazawa University, Kanazawa, Japan; 8 Department of General Medicine, Nagasaki University Hospital, Nagasaki University Graduate School of Biomedical Sciences, Nagasaki, Japan

**Keywords:** Adult-onset Still’s disease, Sepsis, IL-18, FGF-2, Cytokine profile

## Abstract

**Objective:**

To identify potential biomarkers to distinguish adult-onset Still’s disease (AOSD) from sepsis.

**Method:**

We recruited 70 patients diagnosed with AOSD according to the Yamaguchi criteria, 22 patients with sepsis, and 118 age-matched controls. Serum levels of 40 cytokines were analyzed using multi-suspension cytokine array. We performed a cluster analysis of each cytokine in the AOSD and sepsis groups in order to identify specific molecular networks. Further, multivariate classification (random forest analysis) and logistic regression analysis were used to rank the cytokines by their importance and determine specific biomarkers for distinguishing AOSD from sepsis.

**Results:**

Seventeen of the 40 cytokines were found to be suitable for further analyses. The serum levels of eleven were significantly higher in patients with AOSD than healthy controls. Levels of serum fibroblast growth factor 2 **(**FGF-2), vascular endothelial growth factor (VEGF), granulocyte colony-stimulating factor (G-CSF), and interleukin (IL)-18 were significantly elevated in patients with AOSD compared with those with sepsis, and cytokine clustering patterns differed between these two groups. Multivariate classification followed by logistic regression analysis revealed that measurement of both FGF-2 and IL-18 could distinguish AOSD from sepsis with high accuracy (cutoff value for FGF-2 = 36 pg/mL; IL-18 = 543 pg/mL, sensitivity 100%, specificity 72.2%, accuracy 93.8%).

**Conclusion:**

Determination of FGF-2 and IL-18 levels in combination may represent a biomarker for the differential diagnosis of AOSD from sepsis, based on the measurement of multiple cytokines.

## Introduction

Adult-onset Still’s disease (AOSD) is a rare autoinflammatory disease characterized by remittent fever, arthritis, a salmon-pink-colored rash in the febrile stage, leukocytosis, lymphadenopathy, and elevated serum ferritin levels [1]. Although the underlying causes of AOSD are currently unknown at present, autoantibodies are not usually detected, and most of AOSD symptoms can be attributed to hypercytokinemia due to abnormal activation of inflammatory cells related to the innate immune system, including neutrophils, monocytes, and macrophages [2].

There are no disease-specific serological markers for the diagnosis of AOSD, and it is considered representative of diseases causing fever of unknown origin (FUO). Thus, early diagnosis is usually difficult, and delayed diagnosis is associated with poor prognosis. Furthermore, severe cases can worsen because of complication with macrophage activation syndrome (MAS) or disseminated intravascular coagulation (DIC) [3]. Early intervention is therefore necessary after appropriate differentiation, in order to improve patient outcomes.

Sepsis is a clinical syndrome that is caused by a dysregulated host response to infection. The release of excess cytokines by activated innate immune cells, particularly macrophages, plays an important role in the pathogenesis of sepsis [4, 5]. In severe sepsis, such as in AOSD, MAS and DIC are often associated with fatal outcomes owing to multiple organ failure [4]. Thus, some overlap in pathology between sepsis and AOSD may make differentiation clinically difficult, especially in severe cases. A previous report demonstrated that the causative organism is not identified in approximately half of the cases of sepsis [6].

Serum levels of interleukin (IL)-1β, IL-6, and IL-18 have been reported to have utility as serum biomarkers for diagnosis and disease evaluation of AOSD [7–10]. However, these cytokines are elevated in a several other inflammatory diseases, including severe infection, and the clinical manifestations of AOSD are also similar to those observed in severe infection. Notably, differentiation of AOSD from sepsis complicated with DIC or presented hyperferritinemia is a major clinical complication. To address this issue, the present study aimed to identify specific biomarkers to distinguish AOSD from sepsis using multivariate analysis combined with the random forest method based on comprehensive analyses of serum cytokines and chemokines.

## Methods

### Patients and controls

This study was registered with the University Hospital Medical Information Network Clinical Trials Registry [http://www.umin.ac.jp/ctr/] as UMIN000030922. We prospectively recruited consecutive patients with AOSD who were treated at Nagasaki University, Shinshu University, Kanazawa University, and Sasebo City Medical Center between April 2014 and October 2018. The diagnosis of AOSD was based on the Yamaguchi criteria [11]. The diagnosis of MAS was confirmed by the findings of hemophagocytosis in the bone marrow aspiration. We also recruited patients with sepsis who were admitted to the rheumatology department of Nagasaki University Hospital between April 2016 and October 2018 and required differential diagnoses from FUO. These patients did not have any other underlying rheumatological conditions. Sepsis was defined according to the Sepsis-3 criteria: increase in Sequential Organ Failure Assessment score by ≥ 2 at day 1 and suspicion of infection [12]. All participants underwent clinical assessment and provided blood samples for analysis at the time of admission. The control group was recruited from staff at Nagasaki University and residents of the town of Saza in Nagasaki prefecture who underwent specific health checkups in 2016. Inclusion criteria for the control group were no past or present medical histories of inflammatory disease.

All patients provided written informed consent for participation, and the study and all its protocols were approved by the Institutional Review Board of Nagasaki University and related centers (approval no. 18011512-4). Studies involving the residents of Saza were approved by the Ethics Committee for Human Use of Nagasaki University (approval no. 14051404). Written informed consent was obtained from the residents of Saza who underwent specific health checkups.

### Multiplex cytokine and chemokine bead assays

Serum samples were centrifuged at 3000×*g* for 5 min, and the supernatants were collected and stored at − 80 °C for a maximum of 90 days prior to analysis. A blinded multiplex cytokine bead assay was performed in parallel using the Bio-plex MAGPIX™ Human Cytokine assay (Bio-Rad, Hercules, CA, USA) and MILLIPLEX MAP Human Cytokine/Chemokine Magnetic Bead Panel 1-Premixed 38 Plex (Millipore, Billerica, MA, USA) kits, according to the manufacturers’ instructions. Cytokines that were frequently found to be at non-detectable levels were excluded from analysis.

### Statistical analysis

Baseline demographic characteristics and cytokine/chemokine levels of the study population were compared using the Kruskal-Wallis test followed by Dunn’s multiple comparisons test. Correlations between pairs of serum markers were calculated using Spearman’s rank correlation test. To rank the cytokine levels, we performed the multivariate classification algorithm of random forest analysis (RFA) using the R package RandomForest (http://cran.r-project.org/web/packages/randomForest/) version 4.6.12 software, as previously described [13]. We subsequently selected a classifier consisting of a combination of cytokine markers that yielded the best classification performance to predict AOSD by multiple logistic regression analysis. We then calculated the sensitivity, specificity, accuracy, receiver operator characteristic (ROC) curve, area under the curve (AUC), and Akaike’s information criterion (AIC). Statistical analyses were performed using R software (version 3.2.3) and JMP pro (version 13.0) software (SAS Institute, Cary, NC, USA). All reported *p* values are two-sided, and a *p* value of < 0.05 was considered statistically significant. Bonferroni’s correction for multiple-cytokine testing (*n* = 17) was applied, and *p* < 0.00284 was considered significant.

## Results

### Study population

The study population comprised 70 patients with AOSD, 22 with sepsis, and 118 age- and sex-matched healthy controls. Table 1 presents the demographic, clinical, and laboratory characteristics of patients with AOSD and sepsis. All patients had a treatment course of < 1 month, and none of the patients had undergone treatment with glucocorticoids or other immunosuppressive agents. The median ages at diagnosis were 48 years and 59 years in the AOSD and the sepsis groups, respectively.

**Table 1:** The demographic, clinical, and laboratory characteristics of patients with AOSD and sepsis

Characteristic	AOSD patients (*n* = 70)	Sepsis patients (*n* = 22)	Healthy controls (*n* = 118)	*p* value (AOSD vs. sepsis)
Age at diagnosis, years	48 (33–65)	59 (44–71)	56 (47–65)	0.12
Female, *n* (%)	44 (71)	12 (54)	11 (58)	0.17
Ferritin at diagnosis, ng/mL	4019 (1184–11,870)			
CRP at diagnosis, mg/L	87 (48–135)	116 (99–148)		0.056
ESR at diagnosis, mm/h	48 (34–73)			
WBC at diagnosis, /μL	12,290 (6690–17,455)	15,300 (13,625–18,500)		0.12
AST at diagnosis, U/L	50 (31–93)			
ALT at diagnosis, U/L	36 (17–112)			
SOFA score		3 (2.8–4.3)		
DIC, *n* (%)	3 (4)	3 (17)		0.26
MAS, *n* (%)	5 (7)	0 (0)		

*CRP* C-reactive protein, *ESR* erythrocyte sedimentation rate, *WBC* white blood cell, *AST* aspartate aminotransferase, *ALT* alanine animotransferase, *SOFA* Sequential Organ Failure Assessment, *DIC* disseminated intravascular coagulation, *MAS* macrophage activation syndrome

### Cytokine profiles of patients with AOSD, sepsis, and healthy controls

After exclusion of cytokines that were frequently non-detectable, we were able to analyze 17 cytokines: EOTAXIN (CCL11), fibroblast growth factor 2 (FGF-2), basic granulocyte colony-stimulating factor (G-CSF), granulocyte macrophage colony-stimulating factor (GM-CSF), CXCL1 (growth-regulated protein alpha precursor [GRO]), interferon-γ (IFN-γ), IL-17, IL-18, IL-6, IL-8, CXCL10 (interferon gamma-inducible protein 10 [IP-10]), CCL2 (monocyte chemoattractant protein-1 [MCP-1]/MCAF), CCL22 (human macrophage-derived chemokine [MDC]), CCL3 (macrophage inflammatory protein-1a (MIP-1a]), CCL4 (macrophage inflammatory protein-1b [MIP-1b]), TNF-α, and vascular endothelial growth factor (VEGF).

**Fig. 1:**
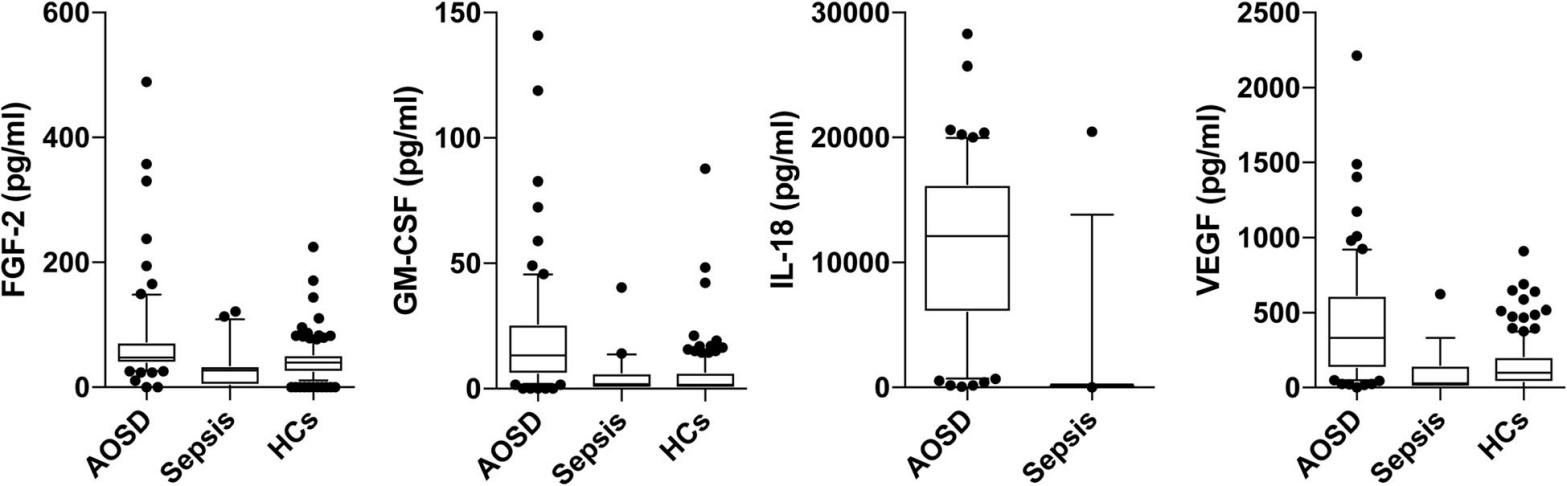
A multiplex cytokine bead assay of FGF-2, GM-CSF, IL-18, and VEGF in the serum of patients with AOSD and sepsis. Data are presented as box and whisker plots [median, interquartile range, and range (10–90 percentiles)]

Serum levels of four cytokines were significantly elevated in patients with AOSD compared with those with sepsis (median FGF-2, 48.7 pg/mL vs. 25.7, *p* < 0.0001; median GM-CSF, 13.1 pg/mL vs. 1.8 pg/mL, *p* < 0.0001; median IL-18, 12,070 pg/mL vs. 104.7 pg/mL, *p* < 0.0001; and median VEGF, 333.3 pg/mL vs. 28.6 pg/mL, *p* < 0.0001) (Fig. 1). Eleven cytokines (FGF-2, G-CSF, GM-CSF, CXCL1, IFN-γ, IL-17, IL-6, IL-8, CXCL10, TNF-α, and VEGF) were significantly increased in the AOSD group compared with the control group (Table 2).

**Table 2:** Cytokine profile of patients with AOSD, those with sepsis, and the healthy controls

Cytokine	HCs (*n* = 118)	AOSD (*n* = 70)	Sepsis (*n* = 18)	*p* value		
				AOSD vs. sepsis	Sepsis vs. HCs	AOSD vs. HCs
EOTAXIN (CCL11)	137.5 (95.1–186.4)	104.0 (66.0–134.0)	123.4 (70.5–172.5)	0.41	0.54	0.01
FGF-2	37.7 (21.2–58.1)	48.7 (41.1–74.4)	25.7 (11.1–34.1)	< 0.0001	0.0057	< 0.0001
G-CSF	8.2 (0.1–22.5)	100.9 (50.5–173.6)	124.1 (45.9–255.2)	0.69	< 0.0001	< 0.0001
GM-CSF	1.5 (0.1–6.7)	13.1 (5.7–26.8)	1.8 (0.1–3.2)	< 0.0001	0.41	< 0.0001
GRO (CXCL1)	978.1 (775.5–1237)	1488 (1034–2383)	697.2 (432.1–1449)	0.027	0.25	< 0.0001
IFN-γ	4.7 (1.5–11.3)	22.7 (7.5–60.2)	12.5 (3.0–21.1)	0.052	0.13	< 0.0001
IL-17	1.9 (0.3–6.2)	5.3 (0.9–18.5)	0.13 (0.1–7.4)	0.019	0.29	0.0002
IL-18	N/A	12,070 (6027–16,451)	104.7 (75.1–382.1)	< 0.0001	N/A	N/A
IL-6	0.1 (0–0.1)	31.7 (12.3–69.7)	28.1 (0.7–100.5)	0.55	< 0.0001	< 0.0001
IL-8	63.0 (28.3–108.3)	30.5 (15.6–50.9)	49.8 (35.7–56.7)	0.96	0.44	< 0.0001
IP-10 (CXCL10)	277.9 (221.0–351.8)	1406 (661.8–3806)	2082 (542.3–4212)	0.81	< 0.0001	< 0.0001
MCP-1 (CCL2)	655.9 (530.5–838.7)	790.8 (475–1675)	1510 (811–3281)	0.13	0.027	0.011
MDC (CCL2)	848.95 (703.0–1008)	668 (338.5–1013)	352.3 (753.7–1013)	0.14	< 0.0001	0.0036
MIP-1a (CCL3)	14.9 (0.1–29.4)	14.2 (1.1–20.1)	18.6 (3.4–35.3)	0.37	0.51	0.42
MIP-1b (CCL4)	59.6 (32.3–82.8)	62.1 (39.6–86.2)	40.3 (9.5–59.0)	0.11	0.16	0.13
TNF-α	12.6 (9.3–16.7)	30.1 (18.1–68.2)	26.9 (16.7–73.9)	0.71	< 0.0001	< 0.0001
VEGF	99.4 (31.9–208.5)	333.3 (128.0–616.4)	28.6 (0.1–151.2)	< 0.0001	0.083	< 0.0001

Values are the median (interquartile range) pg/mL. *p* values were established using the Kruskal-Wallis test followed by a Dunn’s multiple comparisons test. *FGF* fibroblast growth factor, *GM-CSF* granulocyte macrophage colony-stimulating factor, *GRO* growth-regulated protein alpha precursor, *G-CSF* granulocyte colony-stimulating factor, *IL* interleukin, *MCP-1* monocyte chemoattractant protein-1, *MDC* human macrophage-derived chemokine, *TNF-α* tumor necrosis factor-alpha, *VEGF* vascular endothelial growth factor

### Comparison of activated cytokine networks between patients with AOSD and patients with sepsis

To compare activated cytokine networks between patients with AOSD and those with sepsis, we further examined the correlations between serum levels of activated individual cytokines in patients with AOSD and in patients with sepsis. We found significant correlations between IL-17 and IL-8 (*r* = 0.819, *p* < 0.0001), TNF-α and IFN-γ (*r* = 0.640, *p* < 0.0001), VEGF and IL-17 (*r* = 0.585, *p* < 0.0001), FGF-2 and GM-CSF (*r* = 0.5843, *p* < 0.0001), and VEGF and IL-8 (*r* = 0.545, *p* < 0.0001) in patients with AOSD.

**Fig. 2:**
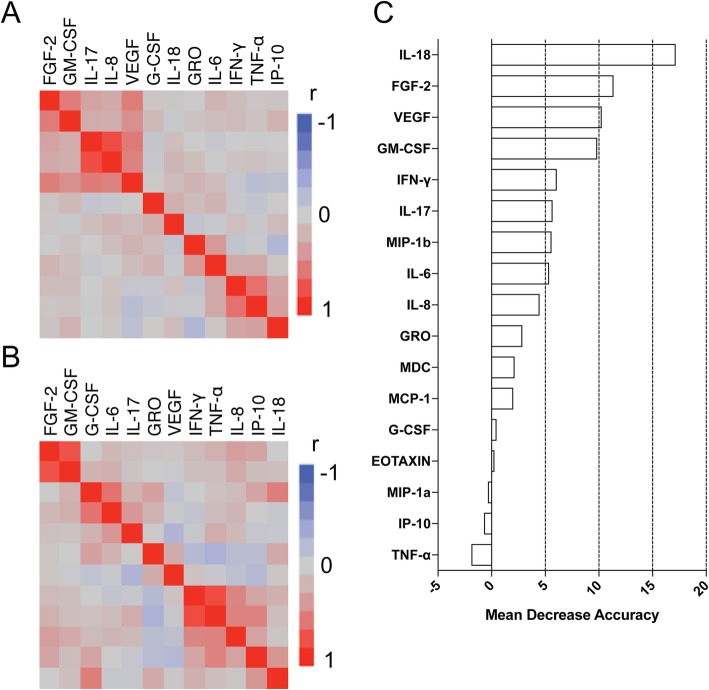
Cytokine networks in the patients with AOSD and sepsis. Hierarchical clustering with a Pearson correlation heatmap of serum cytokine levels among patients with **a** AOSD and **b** sepsis. **c** Cytokines ranked by their relative importance for discriminating AOSD from sepsis. The horizontal axis represents the average decrease in classification accuracy

In the sepsis group, significant correlations were found between IFN-γ and TNF-α (*r* = 0.832, *p* < 0.0001), GM-CSF and FGF-2 (*r* = 0.794, *p* < 0.0001), G-CSF and IL-6 (*r* = 0.626, *p* = 0.0054), G-CSF and IL-18 (*r* = 0.572, *p* = 0.013), IL-8 and TNF-α (*r* = 0.5454, *p* = 0.0192), IP-10 and TNF-α (*r* = 0.537, *p* = 0.022), and IL-8 and IFN-γ (*r* = 0.530, *p* = 0.024). Hierarchical clustering with heatmaps based on the Pearson correlation coefficients is shown in Fig. 2a (for the AOSD group) and Fig. 2b (for the sepsis group).

These results suggest that the cytokine networks in patients with AOSD differ from those in patients with sepsis. Thus, in the AOSD group, FGF-2, GM-CSF, IL-17, IL-18, and VEGF form interrelated networks, whereas in the sepsis group, IFN-γ, TNF-α, IL-8, IL-10, and IL-18 form interrelated networks.

### Identification of combinational biomarkers for the differential diagnosis of AOSD from sepsis by RFA and logistic regression analysis

The results of ranking of cytokines by importance according to RFA are illustrated in Fig. 2c. IL-18 and then FGF-2 were extracted as the most important cytokines for distinguishing AOSD from sepsis (mean decrease accuracy 17.2 and 11.4, respectively). The results of multiple logistic regression analysis and ROC curves for sensitivity, specificity, accuracy, AUC, and AIC are shown in Table 3. The best combination of cytokines to distinguish AOSD from sepsis was found to be IL-18 (> 543 pg/mL) and FGF-2 (> 36 pg/mL), with high accuracy observed (sensitivity 100%, specificity 72.2%, and accuracy 93.8%; Table 3).

**Table 3:** ROC curve in each subset determined by multiple logistic regression analysis

Variables (AOSD vs. sepsis)	Sensitivity	Specificity	Accuracy	AUC	AIC	Cutoff value (pg/mL)
IL-18	93.7	83.3	91.4	0.884	65.67	543
GM-CSF	85.3	83.3	84.9	0.88	61.33	4.4
FGF-2	82.4	88.9	83.7	0.864	62.5	36
VEGF	66.2	88.9	70.9	0.85	70.55	221
**IL-18 > 543 pg/mL + FGF-2 > 36 pg/mL**	**100**	**72.2**	**93.8**	**0.966**	**29.7**	
IL-18 > 543 pg/mL + GM-CSF > 4.4 pg/mL	92.1	83.3	90.1	0.944	37.1	
FGF-2 > 36 pg/mL + GM-CSF > 4.4 pg/mL	89.7	83.3	88.4	0.877	59.32	

Data in boldface indicates the minimum number of cytokines among the subsets. *AIC* Akaike’s information criterion, *AUC* area under the curve, *FGF-2* fibroblast growth factor 2, *GM-CSF* granulocyte macrophage colony-stimulating factor

## Discussion

Infections, malignancies, and systemic rheumatic diseases including AOSD generally account for most cases of FUO. An important aspect in the management of FUO is accurate diagnosis of AOSD, which requires exclusion of infection. It is often difficult to distinguish AOSD from infection because clinical manifestations of AOSD may mimic those of some infections such as sepsis. The similarities between these two diseases include laboratory findings such as marked neutrophilia, increased liver enzymes, or hyperferritinemia. To date, there have been few studies focusing on biomarkers to distinguish the two conditions, and—to the best of our knowledge—there have been no comprehensive analyses involving large numbers of patients with untreated AOSD. The present evaluation of cytokine networks using a multi-suspension cytokine array system resulted in the identification of possible diagnostic biomarkers to distinguish AOSD from sepsis with high accuracy.

Of the biomarkers that were identified, IL-18 is a proinflammatory cytokine belonging to the IL-1 family that induces the production of IFN-γ via inflammasome signals triggered by pathogen-associated or damage-associated molecular patterns (PAMPs or DAMPs) [14]. IL-18 is produced by a variety of cells, including monocytes, macrophages, and dendritic cells [15], and is thought to play a major role in the development of AOSD. In addition to the significant increase in serum levels of IL-18 in patients with AOSD and its correlation with disease activity [9, 16], skin and synovial biopsies from patients with active AOSD showed higher expression levels of IL-18 mRNA compared with controls [9]. In addition, IL-18 has been shown to be overexpressed in other sites, such as lymph nodes and liver, in patients with AOSD [17, 18]. AOSD has been considered an autoinflammatory disease caused by inflammasome activation because of the utility of IL-1 inhibitors [19] and the lack of significant increase of autoantibodies levels [20]. In the pathogenesis of AOSD, IL-18, which is produced by NLPR3 inflammasome activation [21], induces the production of IFN-γ by activating T cells with IL-18 receptors. IL-18 also plays an important role in the development of MAS in patients with AOSD [22].

Accordingly, in contrast with inflammatory cytokines such as IL-1β, IL-6, TNF-α, and IFN-γ, elevation of IL-18 has levels been reported to be characteristic of AOSD [7] and, thus, potentially useful for differentiating AOSD from sepsis [23]. However, the production of IL-18 is increased by lipopolysaccharide-stimulated NLRP3 inflammasome activation [24], indicating that IL-18 levels are increased in patients with sepsis [25]. In line with this observation, serum IL-18 levels of patients with sepsis were elevated to the same levels as in patients with AOSD in some cases in the present study.

Previous studies showed that serum IL-18 levels in patients with AOSD varied widely, from 788 pg/mL (mean value) to 16,327 pg/mL (median value) [7, 9, 16, 17, 23, 26–28]. The median IL-18 level in this study was 12,070 pg/mL, which was higher than that reported in some studies [7, 9, 16, 17, 23, 26, 27]. We speculate that this may be due to differences in the detection range of ELISA and bead array tests as well as differences in patient characteristics. IL-18 levels tended to be higher, especially in patients with AOSD with high disease activity [9, 16], suggesting that high disease activity in the patient population may have influenced these variations.

In contrast to our findings, Rau et al. reported no significant differences in serum IL-18 levels between patients with sepsis and those with AOSD [29]. Although this result differs from our results, a major limitation of that study was the small number of patients in the study cohort (18 patients with AOSD and 14 patients with sepsis). In addition, as the authors emphasize, the wide range of IL-18 levels observed in all patient groups may have biased the statistical analysis. In another study comparing patients with sepsis and AOSD, serum IL-18 levels were higher in patients with AOSD than in those with sepsis [23], which is consistent with our results.

The biological activity of IL-18 is tightly regulated by IL-18 binding protein (IL-18 BP), a natural inhibitor that binds IL-18 with high affinity [30]. Although serum levels of free IL-18 have been shown to be elevated in AOSD and correlate with clinical and biological markers of disease activity [7, 31], we have not been able to accurately assess IL-18 activity because the assay used in this study cannot distinguish free IL-18 that is not bound to IL-18 BP (active) and IL-18 complexed with IL-18 BP (inactive). This is considered to be a limitation of this study.

Although, consistent with previous reports [32], we did not observe serum FGF-2 levels to be elevated in patients with sepsis, no studies have measured serum FGF-2 levels in patients with AOSD. This is therefore the first study to suggest FGF-2 as a new biomarker for AOSD. This cytokine is produced by a number of cell types, including fibroblasts, endothelial cells, and macrophages; it represents the most potent inducer of angiogenesis and is released following tissue injuries and during inflammation [33]. While FGF-2 is induced by IL-1β in human osteoblasts and fibroblasts [34], the cytokine is induced by IL-1β in endothelial cells [35]. Patients with AOSD exhibit synovial hyperplasia and synovitis similar to rheumatoid arthritis (RA) due to the activation of synovial fibroblasts. Elevated serum levels of soluble intercellular adhesion molecule-1 (sICAM-1) have also been reported in the context of AOSD, suggesting that vascular endothelial cells may be activated. Collectively, our findings lead us to speculate that these cells may be the primary source of FGF-2 in patients with AOSD. The most common pathway employed by FGF-2 is the p38 mitogen-activated protein kinase (MAPK) pathway [36], which has been suggested to exacerbate arthritis and bone destruction in RA. Therefore, it is proposed that high levels of FGF-2 may be involved in promoting p38 MAPK-mediated arthritis and inflammatory responses in patients with AOSD.

Our study has several limitations that should be acknowledged. The cross-sectional design and use of serum samples from patients with untreated AOSD mean that continuous serum samples were not used in this study. Although it has been suggested that IL-18 is useful as a biomarker to reflect the therapeutic response and disease activity of AOSD [28, 31, 37] and a phase II clinical trial showed that IL-18 inhibition by administration of recombinant human IL-18BP is effective in the treatment of AOSD [38], further longitudinal studies are needed to verify whether the cytokines identified in this study are associated with therapeutic responses. In addition, the cytokine profiles presented here for patients with sepsis may differ from those of normal populations of patients with sepsis because we included patients admitted for evaluation of FUO who were subsequently diagnosed with sepsis. Therefore, studies involving more patients with sepsis should be conducted in the future. Finally, although the efficacy of an IL-1 inhibitor in patients with severe AOSD has been shown [19], the serum IL-1β level is not high enough for the assay that we used to detect significant differences.

## Conclusions

In conclusion, our study demonstrates that the measurement of FGF-2 and IL-18 levels in combination represents an effective biomarker for the differential diagnosis of AOSD from sepsis. Although differential diagnosis from rheumatic diseases and infectious conditions poses considerable challenges in clinical practice, the findings of this study may help to enhance the diagnostic performance of AOSD in daily practice and drive the discovery of further biomarkers.

## Data Availability

The datasets used and/or analyzed during the present study are available from the corresponding author on reasonable request.

## Abbreviations

AIC: Akaike’s information criterion
AOSD: Adult-onset Still’s disease
AUC: Area under the curve
DIC: Disseminated intravascular coagulation
FGF-2: Fibroblast growth factor 2
FUO: Fever of unknown origin
G-CSF: Granulocyte colony-stimulating factor
GM-CSF: Granulocyte macrophage colony-stimulating factor
IL: Interleukin
MAS: Macrophage activation syndrome
MIP: Macrophage inflammatory protein
RA: Rheumatoid arthritis
RFA: Random forest analysis
ROC: Receiver operator characteristic
VEGF: Vascular endothelial growth factor

## References

[1] Gerfaud-Valentin M, Jamilloux Y, Iwaz J, Seve P (2014). Adult-onset Still’s disease. Autoimmun Rev.

[2] Ohta A, Yamaguchi M, Tsunematsu T, Kasukawa R, Mizushima H, Kashiwagi H, Kashiwazaki S, Tanimoto K, Matsumoto Y, Akizuki M (1990). Adult Still’s disease: a multicenter survey of Japanese patients. J Rheumatol.

[3] Arlet JB, Le TH, Marinho A, Amoura Z, Wechsler B, Papo T, Piette JC (2006). Reactive haemophagocytic syndrome in adult-onset Still’s disease: a report of six patients and a review of the literature. Ann Rheum Dis.

[4] Karakike E, Giamarellos-Bourboulis EJ (2019). Macrophage activation-like syndrome: a distinct entity leading to early death in Sepsis. Front Immunol.

[5] Cinel I, Dellinger RP (2007). Advances in pathogenesis and management of sepsis. Curr Opin Infect Dis.

[6] Gupta S, Sakhuja A, Kumar G, McGrath E, Nanchal RS, Kashani KB (2016). Culture-negative severe sepsis: nationwide trends and outcomes. Chest.

[7] Girard C, Rech J, Brown M, Allali D, Roux-Lombard P, Spertini F, Schiffrin EJ, Schett G, Manger B, Bas S (2016). Elevated serum levels of free interleukin-18 in adult-onset Still’s disease. Rheumatology (Oxford).

[8] Feist E, Mitrovic S, Fautrel B (2018). Mechanisms, biomarkers and targets for adult-onset Still’s disease. Nat Rev Rheumatol.

[9] Chen DY, Lan JL, Lin FJ, Hsieh TY (2004). Proinflammatory cytokine profiles in sera and pathological tissues of patients with active untreated adult onset Still’s disease. J Rheumatol.

[10] Choi JH, Suh CH, Lee YM, Suh YJ, Lee SK, Kim SS, Nahm DH, Park HS (2003). Serum cytokine profiles in patients with adult onset Still’s disease. J Rheumatol.

[11] Yamaguchi M, Ohta A, Tsunematsu T, Kasukawa R, Mizushima Y, Kashiwagi H, Kashiwazaki S, Tanimoto K, Matsumoto Y, Ota T (1992). Preliminary criteria for classification of adult Still’s disease. J Rheumatol.

[12] Singer M, Deutschman CS, Seymour CW, Shankar-Hari M, Annane D, Bauer M, Bellomo R, Bernard GR, Chiche JD, Coopersmith CM (2016). The third international consensus definitions for sepsis and septic shock (Sepsis-3). JAMA.

[13] Koga T, Migita K, Sato S, Umeda M, Nonaka F, Kawashiri SY, Iwamoto N, Ichinose K, Tamai M, Nakamura H (2016). Multiple serum cytokine profiling to identify combinational diagnostic biomarkers in attacks of familial Mediterranean fever. Medicine.

[14] Jamilloux Y, Gerfaud-Valentin M, Martinon F, Belot A, Henry T, Seve P (2015). Pathogenesis of adult-onset Still’s disease: new insights from the juvenile counterpart. Immunol Res.

[15] Dinarello CA, Novick D, Kim S, Kaplanski G (2013). Interleukin-18 and IL-18 binding protein. Front Immunol.

[16] Fujii T, Nojima T, Yasuoka H, Satoh S, Nakamura K, Kuwana M, Suwa A, Hirakata M, Mimori T (2001). Cytokine and immunogenetic profiles in Japanese patients with adult Still’s disease. Association with chronic articular disease. Rheumatology (Oxford).

[17] Conigliaro P, Priori R, Bombardieri M, Alessandri C, Barone F, Pitzalis C, McInnes IB, Valesini G (2009). Lymph node IL-18 expression in adult-onset Still’s disease. Ann Rheum Dis.

[18] Priori R, Barone F, Alessandri C, Colafrancesco S, McInnes IB, Pitzalis C, Valesini G, Bombardieri M (2011). Markedly increased IL-18 liver expression in adult-onset Still’s disease-related hepatitis. Rheumatology (Oxford).

[19] Cavalli G, Tomelleri A, De Luca G, Campochiaro C, Dinarello CA, Baldissera E, Dagna L (2019). Efficacy of canakinumab as first-line biologic agent in adult-onset Still’s disease. Arthritis Res Ther.

[20] Rossi-Semerano L, Kone-Paut I (2012). Is Still’s disease an autoinflammatory syndrome?. Int J Inflam.

[21] Hsieh CW, Chen YM, Lin CC, Tang KT, Chen HH, Hung WT, Lai KL, Chen DY (2017). Elevated expression of the NLRP3 inflammasome and its correlation with disease activity in adult-onset Still disease. J Rheumatol.

[22] Ichida H, Kawaguchi Y, Sugiura T, Takagi K, Katsumata Y, Gono T, Ota Y, Kataoka S, Kawasumi H, Yamanaka H (2014). Clinical manifestations of adult-onset Still’s disease presenting with erosive arthritis: association with low levels of ferritin and Interleukin-18. Arthritis Care Res (Hoboken).

[23] Priori R, Colafrancesco S, Alessandri C, Minniti A, Perricone C, Iaiani G, Palazzo D, Valesini G (2014). Interleukin 18: a biomarker for differential diagnosis between adult-onset Still’s disease and sepsis. J Rheumatol.

[24] Jiang L, Zhang L, Kang K, Fei D, Gong R, Cao Y, Pan S, Zhao M, Zhao M (2016). Resveratrol ameliorates LPS-induced acute lung injury via NLRP3 inflammasome modulation. Biomed Pharmacother.

[25] Grobmyer SR, Lin E, Lowry SF, Rivadeneira DE, Potter S, Barie PS, Nathan CF (2000). Elevation of IL-18 in human sepsis. J Clin Immunol.

[26] Kawaguchi Y, Terajima H, Harigai M, Hara M, Kamatani N (2001). Interleukin-18 as a novel diagnostic marker and indicator of disease severity in adult-onset Still’s disease. Arthritis Rheum.

[27] Chen DY, Lan JL, Lin FJ, Hsieh TY (2005). Association of intercellular adhesion molecule-1 with clinical manifestations and interleukin-18 in patients with active, untreated adult-onset Still’s disease. Arthritis Rheum.

[28] Kudela H, Drynda S, Lux A, Horneff G, Kekow J (2019). Comparative study of Interleukin-18 (IL-18) serum levels in adult onset Still’s disease (AOSD) and systemic onset juvenile idiopathic arthritis (sJIA) and its use as a biomarker for diagnosis and evaluation of disease activity. BMC Rheumatol.

[29] Rau M, Schiller M, Krienke S, Heyder P, Lorenz H, Blank N (2010). Clinical manifestations but not cytokine profiles differentiate adult-onset Still’s disease and sepsis. J Rheumatol.

[30] Novick D, Kim SH, Fantuzzi G, Reznikov LL, Dinarello CA, Rubinstein M (1999). Interleukin-18 binding protein: a novel modulator of the Th1 cytokine response. Immunity.

[31] Jung KH, Kim JJ, Lee JS, Park W, Kim TH, Jun JB, Yoo DH (2014). Interleukin-18 as an efficient marker for remission and follow-up in patients with inactive adult-onset Still’s disease. Scand J Rheumatol.

[32] Faiotto VB, Franci D, Enz Hubert RM, de Souza GR, Fiusa MML, Hounkpe BW, Santos TM, Carvalho-Filho MA, De Paula EV (2017). Circulating levels of the angiogenesis mediators endoglin, HB-EGF, BMP-9 and FGF-2 in patients with severe sepsis and septic shock. J Crit Care.

[33] Shute J, Marshall L, Bodey K, Bush A (2003). Growth factors in cystic fibrosis - when more is not enough. Paediatr Respir Rev.

[34] Sobue T, Zhang X, Florkiewicz RZ, Hurley MM (2001). Interleukin-1 regulates FGF-2 mRNA and localization of FGF-2 protein in human osteoblasts. Biochem Biophys Res Commun.

[35] Lee HT, Lee JG, Na M, Kay EP (2004). FGF-2 induced by interleukin-1 beta through the action of phosphatidylinositol 3-kinase mediates endothelial mesenchymal transformation in corneal endothelial cells. J Biol Chem.

[36] Clark AR, Dean JL (2012). The p38 MAPK pathway in rheumatoid arthritis: a sideways look. Open Rheumatol J.

[37] Colafrancesco S, Priori R, Alessandri C, Perricone C, Pendolino M, Picarelli G, Valesini G (2012). IL-18 serum level in adult onset Still’s disease: a marker of disease activity. Int J Inflam.

[38] Gabay C, Fautrel B, Rech J, Spertini F, Feist E, Kotter I, Hachulla E, Morel J, Schaeverbeke T, Hamidou MA (2018). Open-label, multicentre, dose-escalating phase II clinical trial on the safety and efficacy of tadekinig alfa (IL-18BP) in adult-onset Still’s disease. Ann Rheum Dis.

